# Highly effective and chemoselective hydrodeoxygenation of aromatic alcohols†

**DOI:** 10.1039/d1sc06430d

**Published:** 2022-01-19

**Authors:** Caiyun Xu, Haihong Wu, Zhanrong Zhang, Bingxiao Zheng, Jianxin Zhai, Kaili Zhang, Wei Wu, Xuelei Mei, Mingyuan He, Buxing Han

**Affiliations:** Shanghai Key Laboratory of Green Chemistry and Chemical Processes, School of Chemistry and Molecular Engineering, East China Normal University Shanghai 200062 China hhwu@chem.ecnu.edu.cn hemingyuan@126.com hanbx@iccas.ac.cn; Beijing National Laboratory for Molecular Sciences, CAS Key Laboratory of Colloid and Interface and Thermodynamics, CAS Research/Education Center for Excellence in Molecular Sciences, Institute of Chemistry, Chinese Academy of Sciences Beijing 100190 China zhangzhanrong@iccas.ac.cn hanbx@iccas.ac.cn

## Abstract

Effective hydrodeoxygenation (HDO) of aromatic alcohols is very attractive in both conventional organic synthesis and upgrading of biomass-derived molecules, but the selectivity of this reaction is usually low because of the competitive hydrogenation of the unsaturated aromatic ring and the hydroxyl group. The high activity of noble metal-based catalysts often leads to undesired side reactions (*e.g.*, saturation of the aromatic ring) and excessive hydrogen consumption. Non-noble metal-based catalysts suffer from unsatisfied activity and selectivity and often require harsh reaction conditions. Herein, for the first time, we report chemoselective HDO of various aromatic alcohols with excellent selectivity, using porous carbon–nitrogen hybrid material-supported Co catalysts. The C–OH bonds were selectively cleaved while leaving the aromatic moiety intact, and in most cases the yields of targeted compounds reached above 99% and the catalyst could be readily recycled. Nitrogen doping on the carbon skeleton of the catalyst support (C–N matrix) significantly improved the yield of the targeted product. The presence of large pores and a high surface area also improved the catalyst efficiency. This work opens the way for efficient and selective HDO reactions of aromatic alcohols using non-noble metal catalysts.

## Abstract 2

Porous carbon–nitrogen hybrid material-supported Co catalysts can effectively promote the chemoselective hydrodeoxygenation reaction of a various of aromatic alcohols in ethanol and hydrogen atmosphere, under relatively mild conditions.

## Graphical abstract

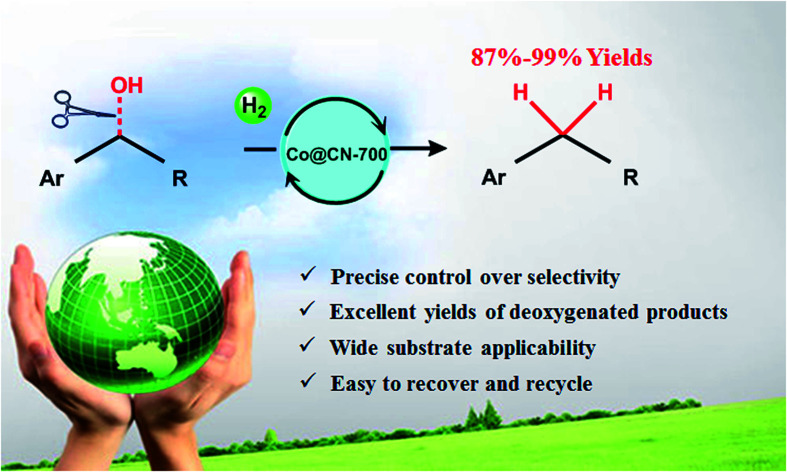

## Introduction

Biomass represents a green and sustainable carbon resource.^1^ In comparison with other energy sources, biomass is renewable, abundant and widely distributed worldwide.^2,3^ Thus, making innovative use of biomass to replace non-renewable fossil reserves for the production of valuable chemical products has attracted significant attention.^4–9^ In this regard, chemical transformation and upgrading of biomass-derived molecules into useful chemical and fuel products are highly important.^10–14^ Owing to the high oxygen content of biomass and its chemical derivatives, the applications of biorefinery products are often limited. For example, bio-oil produced by rapid pyrolysis of biomass is a valuable second-generation renewable energy and resource carrier.^15,16^ However, it generally contains a large number of oxygen-containing compounds, which are thermodynamically unstable. The many different self- and/or cross-polymerization reactions between ketones, aldehydes, acids and alcohols that are contained in bio-oil significantly hinder the incorporation of bio-oil as the feedstock into existing refinery equipment.^17^ To address this problem, many hydrodeoxygenation (HDO) approaches have been developed for the upgrading of biomass-derived molecules.^18^ HDO refers to the transformation of a hydroxyl group into the corresponding alkyl group, which constitutes a powerful synthetic tool for the synthesis of complex natural products, bioactive molecules and petrochemicals.^19,20^ In recent years, chemoselective HDO has not only received extensive attention in organic synthesis, but also has been proved to be an effective way for the upgrading of biomass-derived molecules. Previous studies have also demonstrated that selective HDO is an effective method to improve the stability of bio-oil. To date, researchers have developed many methods for chemoselective HDO of oxygen-containing molecules, such as alcohols, aldehydes, ketones, amides and nitrogen-containing compounds.^21–23^ Among them, alcohols are widely used as versatile precursors for the production of other organic molecules in the chemical industry. The selective HDO of alcohols is one of the most fundamental transformations in organic chemistry and plays a crucial role in the total synthesis of complex natural products bearing multifunctional groups.^24,25^ In terms of biomass upgrading, selective HDO of aromatic alcohols *via* catalytic hydrogenolysis of C–OH bonds is very important, to produce valuable chemicals from lignin which is the only natural renewable aromatic biomass resource. Herein, one of the key challenges is the precise control of selectivity.^26^ The selectivity of HDO of aromatic alcohol substrates is regulated by the competitive hydrogenation of the unsaturated aromatic ring and the hydroxyl group. During the reaction, aromatic alcohols are readily adsorbed on the metal surface of catalysts. Thermodynamically, conformation between the benzene ring and the metal surface tends to be flat. Thus, the π-electron interaction promotes the hydrogenation of the unsaturated aromatic ring, which renders the selectivity of HDO not satisfactory.^27^

Indeed, hydrogenolysis of C–OH bonds has been investigated using both noble and non-noble metal-based catalysts.^28–30^ Noble metal-based catalysts (*e.g.*, Ru,^31,32^ Au^33^ and Pd-based catalysts^34,35^) often exhibit higher activity towards hydrogenation and HDO reactions than their non-noble metal-based counterparts. However, their high activity often inevitably leads to undesired side reactions (*e.g.*, saturation of the aromatic ring) and high hydrogen consumption, which often renders the reaction selectivity not satisfactory. In comparison, less attention has been paid to chemoselective HDO of aromatic alcohols using non-noble metal-based catalysts.^36–38^ The lower activity of non-noble metal catalysts often leads to a high reaction temperature and pressure, which in turn probably deactivates the catalyst. Moreover, a limited range of substrates and unclear active sites under these conditions limit their practical applicability.^39^ In this respect, development of effective, robust, non-noble metal-based heterogeneous catalysts which could be readily separated and recycled is very important and attractive.

Metal organic frameworks (MOFs) can be used as a precursor for the preparation of nanoporous carbon-based materials for different applications, such as catalysis, drug delivery, adsorption and separation.^40–44^ Their large internal surface area, high porosity and mild synthesis conditions make them ideal starting materials for preparation of catalyst supports. Among them, zeolitic imidazolate framework-67 (ZIF-67), a sodalite-type MOF produced by the regular arrangement of Co^2+^ and 2-methylimidazole (2-MeI) is a chemically and thermally stable MOF that has attracted significant attention. Owing to its high amount of active cobalt sites, ZIF-67 is a promising candidate for the synthesis of Co-based catalysts.^45–49^ Direct reduction of ZIF-67 in H_2_ at a high temperature can produce a porous nitrogen-doped carbon supported Co catalyst. During reduction, the Co ions in ZIF-67 are transformed into metallic Co, while the 2-MeI organic linkers become the N-doped carbon support. In this process, ZIF-67 plays a parallel role as both the template and precursor to produce the catalyst.

Herein, for the first time, we report the efficient and chemoselective HDO of aromatic alcohols, using ZIF-67-derived catalysts consisting of Co particles embedded in a porous carbon–nitrogen hybrid material (Co@CN) as the catalyst. Specifically, we synthesized several Co@CN catalysts including Co@CN-500, Co@CN-600 and Co@CN-700, obtained by reducing ZIF-67 at 500, 600 and 700 °C, respectively. In addition, we also synthesized a Co@CN-ph catalyst in a similar way to that of Co@CN-700, by using 1,10-phenanthroline as the organic ligand instead of 2-MeI in the ZIF precursor. The C–OH bonds of both primary and secondary aromatic alcohols are selectively cleaved using Co@CN-700, while the aromatic functionality remains intact. The corresponding aromatic compounds are produced in excellent yields. This efficient chemical transformation could be achieved under mild reaction conditions (120 °C, 2 MPa H_2_). Notably, Co@CN-700 is mesoporous and exhibits a high surface area, and the Co NPs are homogeneously distributed on the carbon–nitrogen hybrid support. These properties lead to the excellent catalytic properties. The catalyst is also magnetically-separable and stable during recycling, making it a very promising catalyst for chemoselective HDO reactions of different types of aromatic alcohols.

## Results and discussion

**Fig. 1:**
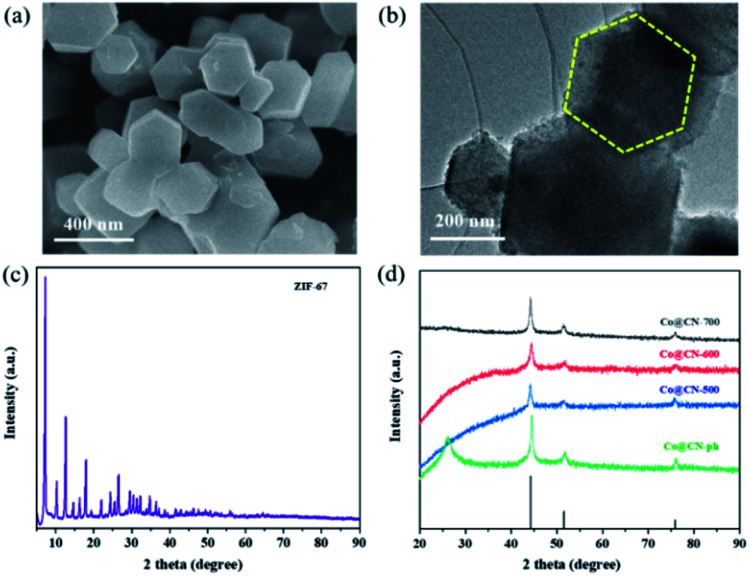
(a) SEM image of Co-ZIF, (b) HR-TEM image of Co-ZIF, (c) XRD pattern of Co-ZIF, (d) XRD patterns of Co@CN-500, Co@CN-600, Co@CN-700 and Co@CN-ph.

Initially, Co-ZIF synthesized at room temperature exhibits high crystallinity and has a uniform polyhedral structure with a smooth surface and diameters between 300 and 500 nm, as revealed by its SEM (Fig. 1a) and high-resolution TEM images (Fig. 1b). The original ZIF-67 shows a representative XRD pattern due to its well-organized framework, which is in good consistency with previous work (Fig. 1c). Then the synthesized Co-ZIF is reduced at 500, 600 and 700 °C, in an hydrogen/argon atmosphere. After reduction, only the diffraction peaks of metallic Co and graphitic carbon are present in the XRD patterns, indicating the successful transformation of Co-ZIF into Co@CN (Fig. 1d). With the increase of the reduction temperature, the diffraction peaks of Co become more dominant, due to increased crystallinity of Co particles.

**Fig. 2:**
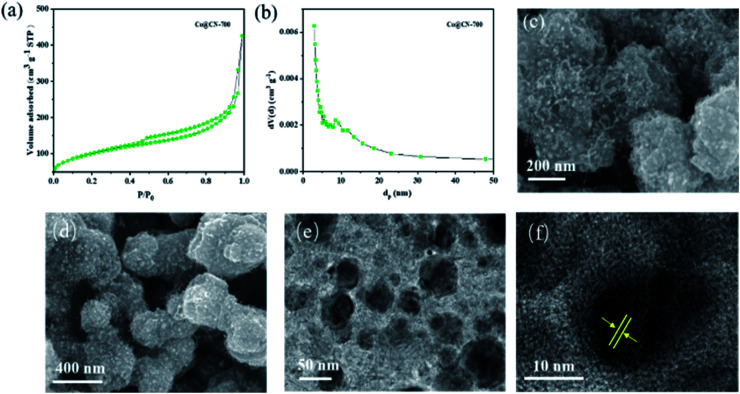
Characterization results of the Co@CN-700 catalyst. (a) N_2_-adsorption–desorption isotherm, (b) BJH pore size distribution, (c) and (d) SEM images, (e) TEM image, and (f) corresponding lattice fringes of cobalt nanoparticles.

The N_2_ adsorption–desorption isotherm and BJH pore size distribution of Co@CN-700 indicates that it exhibits a mesoporous structure (Fig. 2). When *P*/*P*_0_ < 0.2, the adsorption speed of the catalyst is fast. When *P*/*P*_0_ is between 0.4 and 0.8, the catalyst has obvious hysteresis, indicating the presence of mesopores. When *P*/*P*_0_ is close to 1, the isotherm does not tend to be flat but adsorbs upwards, suggesting the presence of large pores. Notably, the BET surface area of Co@CN catalysts increased from 48.9 m^2^ g^−1^ to 111.9 m^2^ g^−1^ with the increase of the reduction temperature from 500 °C to 700 °C. For more structural details, please refer to the ESI (Fig. S1 and Table S1†). SEM images of Co@CN-700 (Fig. 2c and d) revealed that after reducing Co-ZIF, the polyhedron morphology of ZIF crystals changed into rough and spongy-type particles. The Co nanoparticles are uniformly dispersed in the Co@CN-700 catalyst and the interplanar distance of the Co nanoparticles for the lattice fringe is around 0.12 nm, as shown in the HR-TEM image of the Co@CN-700 catalyst (Fig. 2f). More morphological details are shown in the ESI (Fig. S2†). ICP-OES analysis indicates that the Co content in Co@CN-700 is about 38 wt% (ESI, Table S2†).

**Fig. 3:**
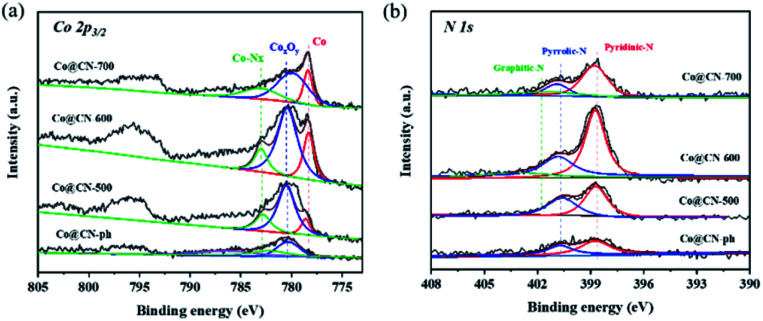
Deconvoluted XPS core-level spectra of Co@CN catalysts. (a) Co 2p core level peaks, and (b) N 1s core level peaks.

XPS characterization of synthesized catalysts was further conducted to investigate their chemical structures and bonding states (Fig. 3). The high-resolution Co 2p_3/2_ XPS spectra were deconvoluted into three distinct peaks centered at 778.1 eV, 780.0 eV and 783.0 eV, which are assigned to Co^0^, Co_*x*_O_*y*_ and Co-N_*x*_, respectively. The intensity of the characteristic peaks of Co_*x*_O_*y*_ (*ca.* 780.0 eV) and Co-N_*x*_ (*ca.* 783.0 eV) decreased with the increase of the reduction temperature from 500 to 700 °C. This indicates that Co_*x*_O_*y*_ and Co-N_*x*_ are reduced to Co^0^ during reduction. The characteristic peak of Co^0^ is the most dominant in the XPS spectrum of Co@CN-700. In contrary, the characteristic peak of Co_*x*_O_*y*_ in the XPS spectrum of Co@CN-ph is the most significant, while the Co-N_*x*_ and Co^0^ diffraction peaks are not shown. Hence, the presence of Co-N_*x*_ could potentially promote the formation of Co^0^ (Fig. 3a). The N 1s spectrum was deconvoluted into three sub-peaks, corresponding to pyridinic-N at 398.4 eV, pyrrolic-N at 400.3 eV and graphitic-N at 401.4 eV (Fig. 3b). The intensity of graphite-N slightly increased with the increase of the reduction temperature from 500 °C to 700 °C, because of its higher thermal stability. No graphitic-N was observed from the XPS spectrum of Co@CN-ph.

Subsequently, we started our investigation using 1-phenylethanol as a model compound under a hydrogen atmosphere. Using ethanol as the solvent, the control experiment indicated that the reaction could not proceed at 120 °C in the absence of the catalyst (Table 1, entry 1). Commercial Co_3_O_4_ and Zn@CN synthesized in a similar way to that of Co@CN as catalysts could not catalyze the reaction as well (Table 1, entry 4 and 5). In contrast, the Co@CN catalysts synthesized by ZIF-67 at different temperatures including Co@CN-500, Co@CN-600 and Co@CN-700 could significantly promote the reaction (Table 1, entries 8–10). Co@CN-700 showed the best catalytic performance and the C–OH bond of 1-phenylethanol was effectively and selectively cleaved, affording ethylbenzene with a yield of up to 99.7% (Table 1, entry 10). Using other common solvents such as THF and cyclohexane instead of ethanol did not significantly affect the yields of the targeted product, although slightly lower yields of ethylbenzene were obtained (Table 1, entry 11 and 12). Activated carbon supported Co nanoparticles (Co@AC) and Co@CN-ph synthesized in a similar way to that of Co@CN-700 could also promote the reaction, and the yields of ethylbenzene reached 90.8% (Table 1, entry 6) and 71.8% (Table 1, entry 7), respectively. These catalysts were less effective in comparison with Co@CN-700. The abovementioned experimental results clearly suggest that the structure of the N-doped carbon skeleton had a significant effect on the reactivity of the HDO reaction. This is primarily because the incorporation of nitrogen atoms (*i.e.*, formation of the carbon–nitrogen hybrid) changed the chemical, electrical and functional properties of the carbon matrix, thereby affecting the catalytic activity of the loaded metal NPs. This effect is also verified by the XPS characterization that the Co-N_*x*_ peak is very obvious in Co@CN materials, but almost missing in the spectrum of Co@CN-ph. The strong interaction between nitrogen atoms and Co nanoparticles not only stabilized the Co particles, but also improved the reducing ability of the catalyst, enabling the alcohol groups to be efficiently converted into the alkyl group. For more details on optimization of reaction conditions, please refer to the ESI (Fig. S3†).

**Table 1:** Selective HDO reaction of 1-phenylethanol

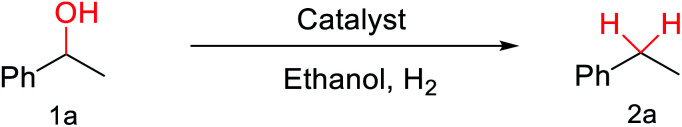					
Entry	Catalyst	Solvent	H_2_ (MPa)	Temp. (°C)	Yield (%)
1	Blank^a^	Ethanol	2	120	n.r.
2	ZIF-67	Ethanol	2	120	n.r.
3	ZIF-67 (air)^b^	Ethanol	2	120	n.r.
4	Co_3_O_4_	Ethanol	2	120	n.r.
5	Zn@CN	Ethanol	2	120	n.r.
6	Co@AC	Ethanol	2	120	90.8
7	Co@CN-ph^c^	Ethanol	2	120	71.8
8	Co@CN-500^dd^	Ethanol	2	120	90.4
9	Co@CN-600^e^	Ethanol	2	120	97.2
10	Co@CN-700^f^	Ethanol	2	120	99.7
11	Co@CN-700	THF	2	120	96.7
12	Co@CN-700	Cyclohexane	2	120	93.2
13	Co@CN-700	Ethanol	2	100	45.1
14	Co@CN-700	Ethanol	1	120	92.5
15	Co@CN-700 (20 mg)	Ethanol	2	120	56.2

a Without catalyst.

b ZIF-67 was calcined in air at 700 °C.

c 1,10-Phenanthroline was used instead of MeI as the ligand for synthesizing the catalyst precursor.

d ZIF-67 was reduced at 500 °C in 10% H_2_/Ar.

e ZIF-67 was reduced at 600 °C in 10% H_2_/Ar.

f ZIF-67 was reduced at 700 °C in 10% H_2_/Ar. Reaction conditions: 1-phenylethanol (1 mmol), catalyst (40 mg), solvent (2 mL), reaction time: 8 h. The GC yields were obtained using dodecane as an internal standard.

After optimization of reaction conditions, we further explored the substrate scope for the HDO reaction over Co@CN-700. The C–OH bonds of a diverse range of secondary and primary alcohols were selectively and effectively cleaved, affording the corresponding aromatic hydrocarbons in excellent yields (Table 2). Specifically, phenylethanol derivatives with various electron-donating or electron-withdrawing substituent groups on the aryl ring (1b–1k) can be effectively converted into dehydroxylated products with excellent yields (87.8–99%). Among them, several substrates (*e.g.*, 1f, 1g, 1i, and 1q) which contain hydroxyl and methoxy groups on the benzene ring can be obtained from lignin transformation. Hence, the designed selective HDO catalyst is also very suitable for further upgrading of biomass-derived molecules. Moreover, we have also tested several bio-based aldehydes that could be directly derived from lignin and cellulose (*e.g.*, 1u, 1v, and 1w), and their corresponding products were generated in excellent yields. Secondary alcohols with various carbon chain lengths (1l–1n) are also suitable substrates, and the corresponding selective HDO products were generated in excellent yields. Moreover, the designed catalyst is effective for selective HDO of monohydric alcohol substrates with excellent selectivity (1o–1t). For comparison, we also tested ethanol and other aliphatic alcohol substrates with various chain lengths including *n*-butanol, *n*-pentanol, *n*-hexanol and *n*-octanol, and these aliphatic alcohols cannot be converted under the same conditions using Co@CN-700. The hydroxyl groups in these substrates are intact after the reaction (Fig. S4†). Hence, the solvent is stable under reaction conditions and the presence of aromatic functionality (*i.e.*, aryl group) is crucial for the HDO reaction to proceed.

**Table 2:** Substrate scope of the chemoselective HDO reaction^a^

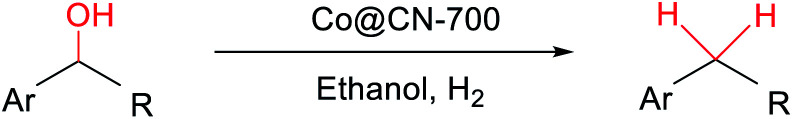					
Substrate	Product	Yield	Substrate	Product	Yield
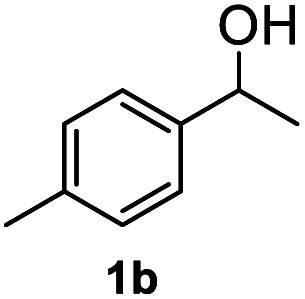	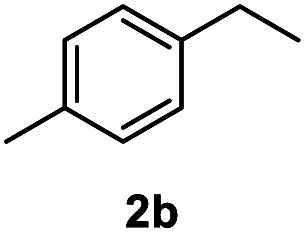	91.7%	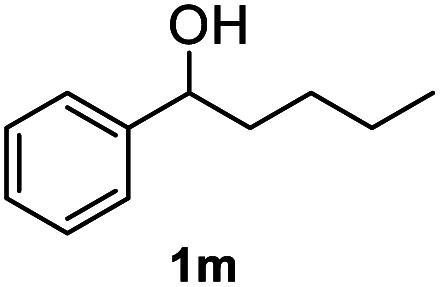	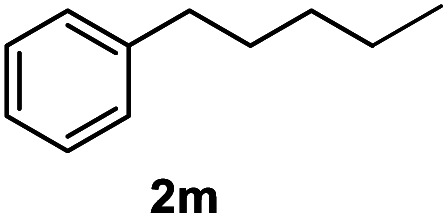	>99%
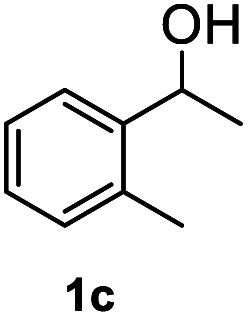	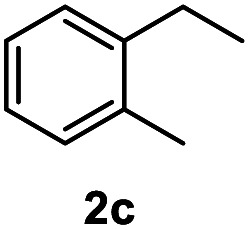	>99%	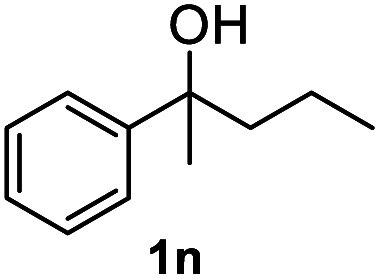	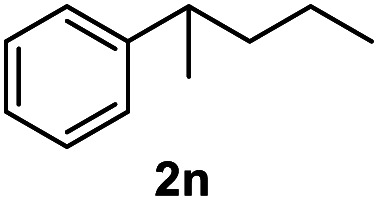	>99%
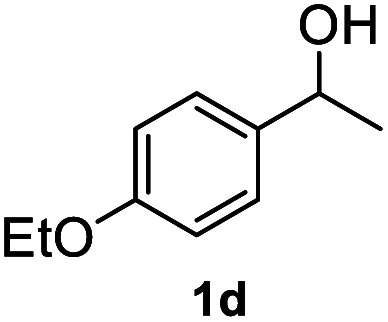	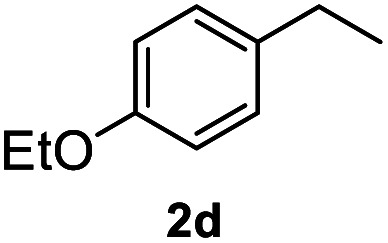	>99%	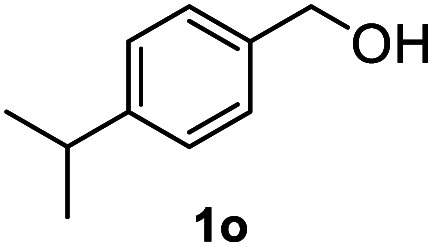	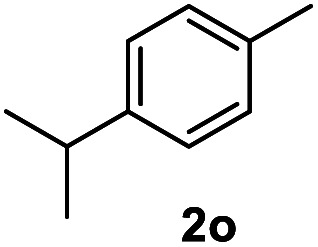	91.8%
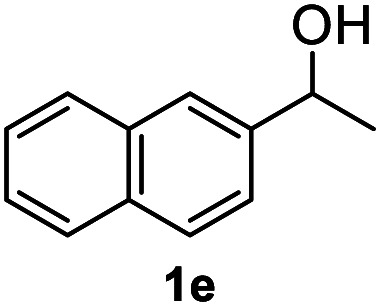	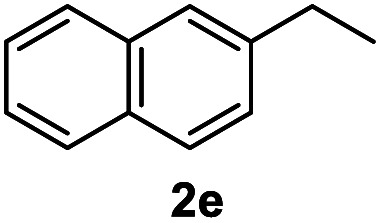	87.8%	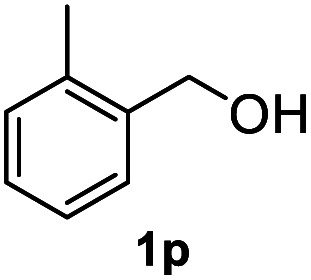	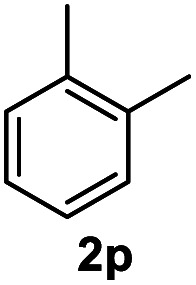	>99%
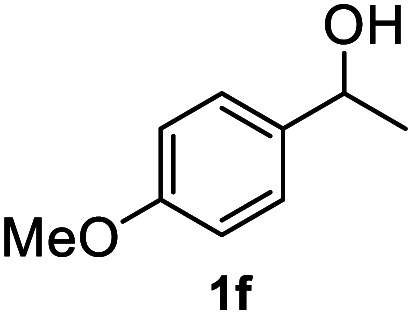	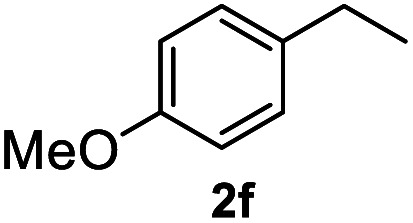	>99%	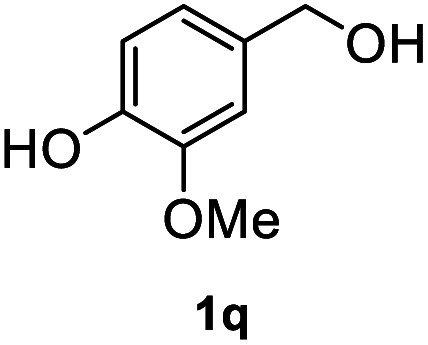	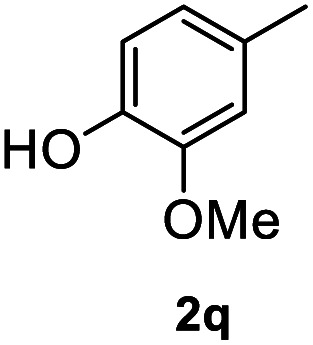	>99%
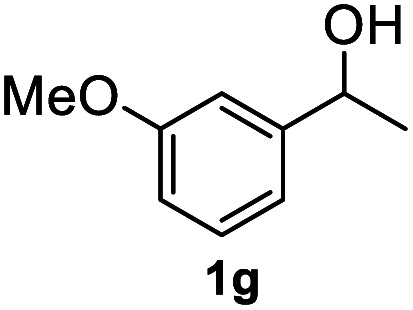	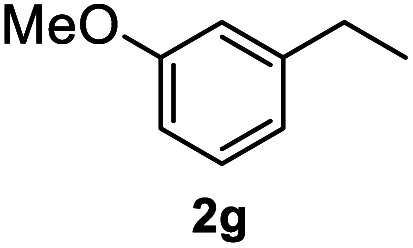	>99%	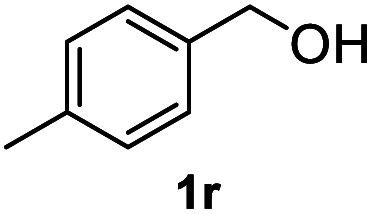	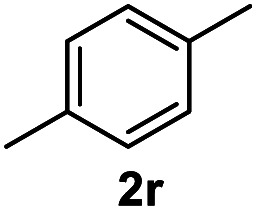	>99%
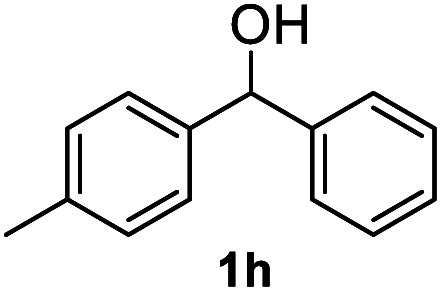	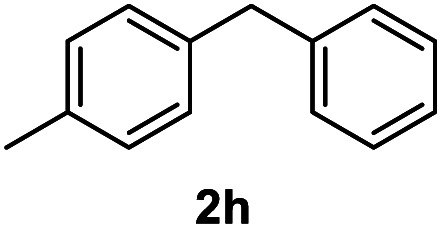	>99%	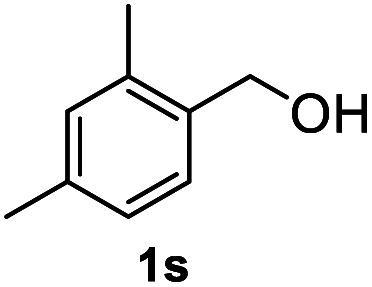	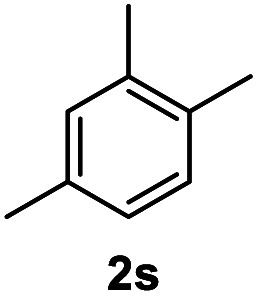	>99%
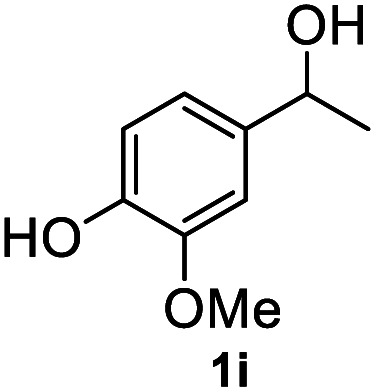	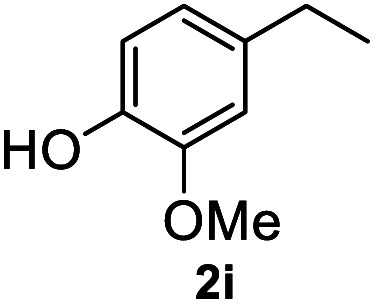	>99%	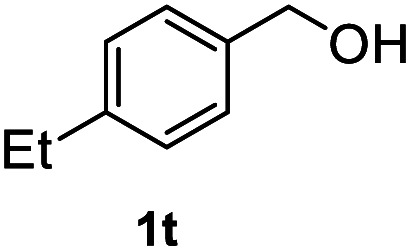	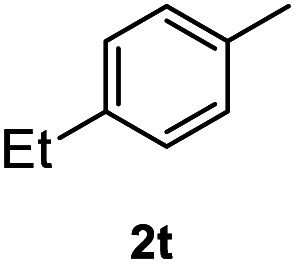	>99%
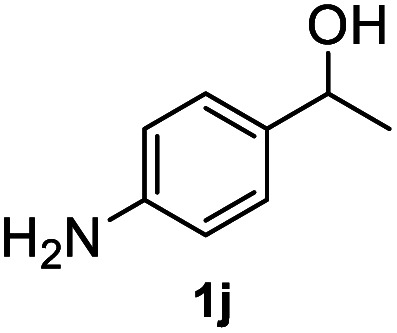	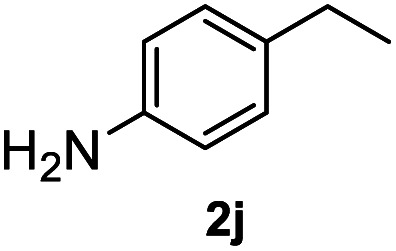	94.1%	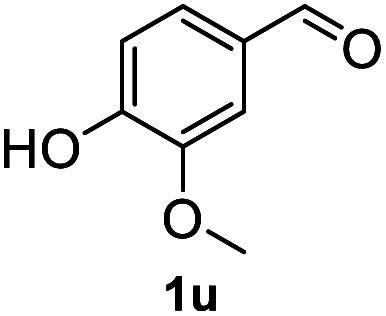	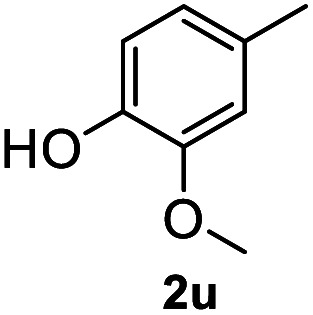	>99%
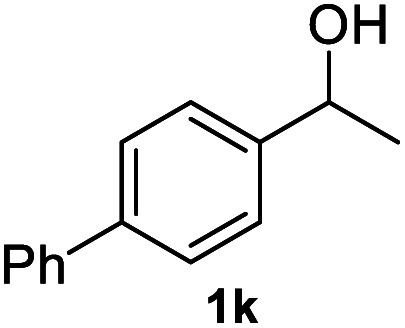	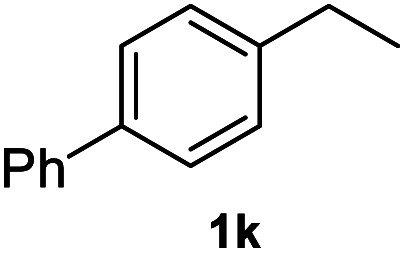	93.1%	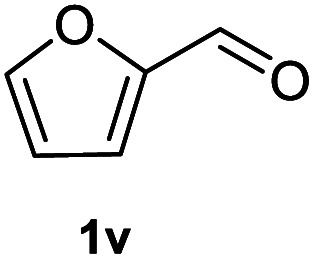	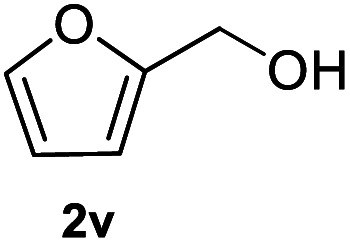	>99%
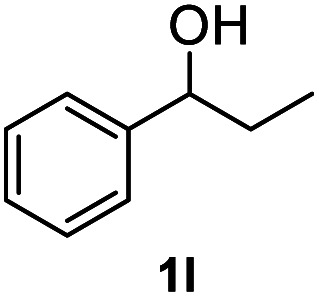	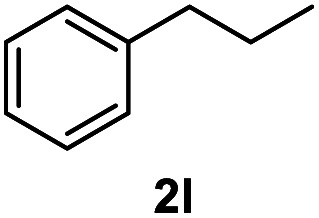	>99%	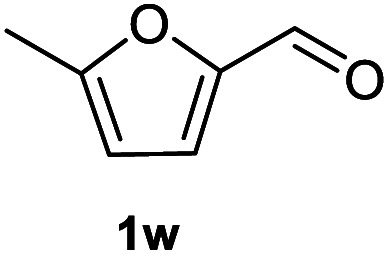	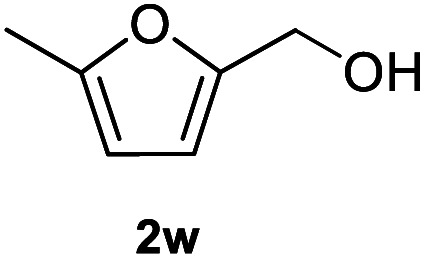	90.3%

a Unless otherwise noted, the reaction was carried out with 1 mmol substrate, 2 mL ethanol, and 40 mg catalyst (Co@CN-700). Reaction temperature: 120 °C, reaction time: 8 h, and H_2_ pressure: 2 MPa.

**Fig. 4:**
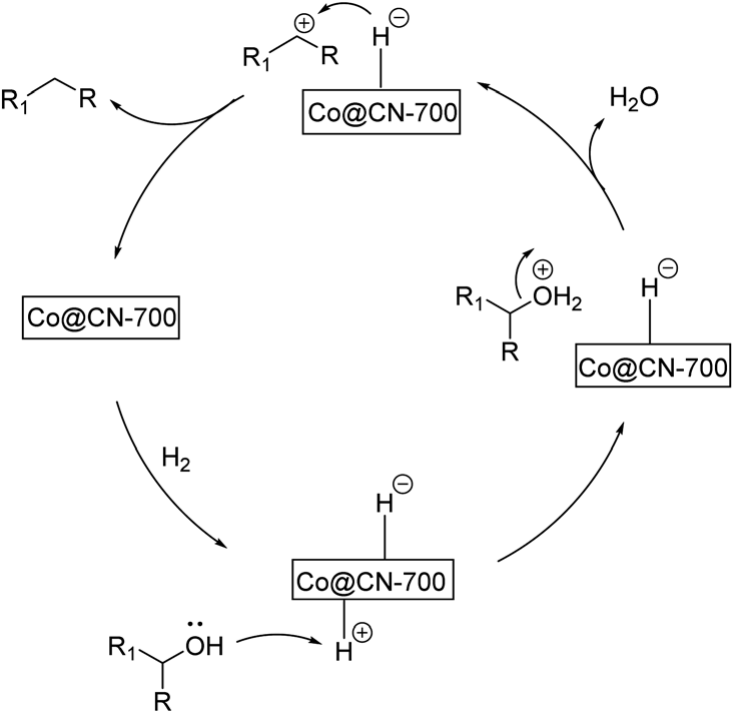
Possible reaction pathway for the selective HDO reaction.

Based on the above-mentioned experimental results, we propose the following reaction pathway for the selective HDO reaction (Fig. 4). First, both aromatic alcohol substrates and H_2_ molecules are adsorbed onto the catalyst. The catalyst promotes the hetero-cracking of H_2_ molecules to generate H^+^ and H^−^. Then, H^+^ combines with the hydroxyl group of the alcohol substrate and a water molecule is released. The resultant benzyl cations (Ph-CH_2_^+^ or Ph-CH^+^-R) can be stabilized by aromatic rings. This effect is more obvious for substrates with the electron-donating groups on the benzene ring. Finally, H^−^ is attached to the benzyl cation to generate the final aromatic alkane product. As abovementioned, the XPS characterization results indicate that Co_*x*_O_*y*_ was primarily transformed into Co^0^ in the Co@CN-700 catalyst. The presence of Co^0^ represents that the active sites of the catalyst are crucial to promote the chemoselective HDO reaction. More importantly, the interaction between nitrogen atoms and Co NPs (Co-N_*x*_ coordination) significantly improves the catalytic performance of Co@CN-700.

**Fig. 5:**
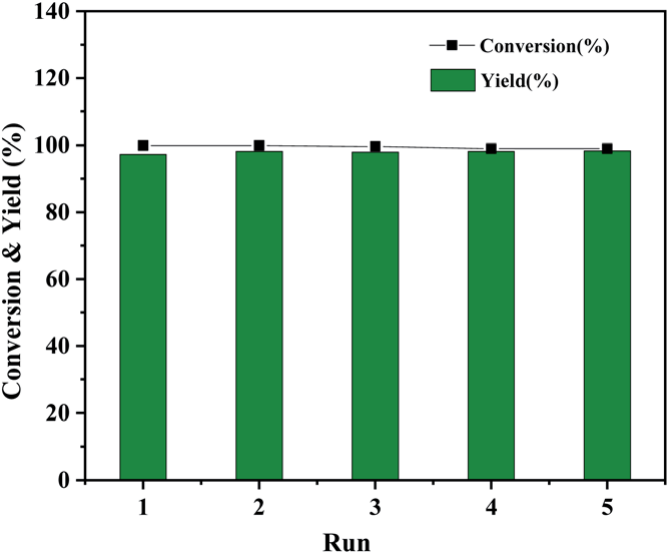
Recyclability test of Co@CN-700. Reaction conditions were the same as those shown in Table 1, entry 1.

The Co@CN-700 catalyst could also be readily recycled under standard conditions. In total, five cycles of the HDO reaction of 1-phenylethanol were carried out at 120 °C (Fig. 5). The spent catalyst could be readily separated from the reaction mixture using an external magnetic field after each reaction (Fig. S5†). The recovered catalyst was washed, dried at 80 °C and reused in the next reaction. The catalyst is still very effective after five runs of the reaction, and the conversion and yield of 1-phenylethanol were almost the same as those of the first run (Fig. 5). In addition, from the detailed morphology and XRD characterization of the recovered Co@CN-700 catalyst, there is no obvious change in its features such as the morphology (Fig. S6 and S7†). XPS characterization of the fresh and recovered Co@CN-700 catalyst indicated that different cobalt species did not change obviously after the reaction (Fig. S8†). The characteristic peak of Co^0^ was still the most dominant in the XPS spectrum of the recovered catalyst, which further proves the excellent recyclability of the synthesized Co@CN-700 catalyst. This also verified that in the case of nitrogen doping, the oxidation resistance of Co^0^ has been improved. As a result, the Co@CN-700 catalyst showed excellent heterogeneous catalytic performance in terms of selectivity, stability and recyclability.

## Conclusions

In summary, the robust Co-ZIF derived Co@CN-700 catalyst can effectively promote the chemoselective HDO reaction of various aromatic alcohols in ethanol and H_2_, under relatively mild conditions (120 °C, 2 MPa H_2_, 8 h). The C–OH bonds of a range of primary and secondary aromatic alcohols were selectively cleaved, while leaving the aromatic functionality intact. Notably, nitrogen atom doping on the carbon skeleton can interfere with the electrical neutrality of adjacent carbon atoms, and this effect leads to imbalance in the charge state, which could enhance the efficiency of the HDO reaction.^48,50^ The interaction/coordination between nitrogen atoms and Co NPs (Co-N_*x*_) is crucial to achieve excellent catalytic efficiency and selectivity. In addition, the presence of large pores and a high surface area also improves the catalytic performance of Co@CN-700. More active sites (*i.e.*, Co^0^) are exposed in this catalyst and thus significantly promote the reaction. During the HDO reaction, water was generated as the only by-product and the catalyst can also be readily recovered and recycled. This robust and cheap catalyst has significant potential application in biofuel and biorefinery processes.

## Data availability

The authors declare that all data supporting the findings of this study are available within the paper [and its ESI†].

## Author contributions

C. Y. X., H. H. W., Z. R. Z., M. Y. H. and B. X. H. conceived the project, and designed and implemented the experiments. C. Y. X., H. H. W., Z. R. Z., M. Y. H. and B. X. H. analyzed the data and wrote the manuscript. All the other authors discussed the results and helped in data analysis.

## Conflicts of interest

The authors declare no competing interests.

## Supplementary Material

SC-013-D1SC06430D-s001

## References

[cit1] Mulvihill M. J., Beach E. S., Zimmerman J. B., Anastas P. T. (2011). Annu. Rev. Energy.

[cit2] Zhou L., Liu Z., Bai Y., Lu T., Yang X., Xu J. (2016). J. Energy Chem..

[cit3] Lam C. H., Bloomfield A. J., Anastas P. T. (2017). Green Chem..

[cit4] Ding W. T., Li H., Zong R., Jiang J. Y., Tang X. F. (2021). ACS Sustainable Chem. Eng..

[cit5] Ma J., Shi S., Jia X., Xia F., Ma H., Gao J., Xu J. (2019). J. Energy Chem..

[cit6] Anutrasakda W., Eiamsantipaisarn K., Jiraroj D., Phasuk A., Tuntulani T., Liu H., Tungasmita D. (2019). Catalysts.

[cit7] Li J., Zhang S., Gao B., Yang A., Wang Z., Xia Y., Liu H. (2016). Fuel.

[cit8] Li J., Liu L., Liu Y., Li M., Zhu Y., Liu H., Kou Y., Zhang J., Han Y., Ma D. (2014). Energy Environ. Sci..

[cit9] Zhang Z., Song J., Han B. (2017). Chem. Rev..

[cit10] Bu Q., Lei H., Zacher A. H., Wang L., Ren S., Liang J., Wei Y., Liu Y., Tang J., Zhang Q., Ruan R. (2012). Bioresour. Technol..

[cit11] Jing Y., Guo Y., Xia Q., Liu X., Wang Y. (2019). Chem.

[cit12] Liu Y., Zhu Y., Li C. (2017). Front. Chem. Sci. Eng..

[cit13] Hu M., Wu W., Jiang H. (2019). ChemSusChem.

[cit14] Lu R., Lu F., Si X., Jiang H., Huang Q., Yu W., Kong X., Xu J. (2018). ChemSusChem.

[cit15] Cherubini F. (2010). Energy Convers. Manage..

[cit16] Pei X., Deng Y., Li Y., Huang Y., Yuan K., Lee J. F., Chan T. S., Zhou J., Lei A., Zhang L. (2018). Nanoscale.

[cit17] Moos G., Emondts M., Bordet A., Leitner W. (2020). Angew. Chem., Int. Ed..

[cit18] Jing Y., Wang Y., Furukawa S., Xia J., Sun C., Hulsey M. J., Wang H., Guo Y., Liu X., Yan N. (2021). Angew. Chem., Int. Ed..

[cit19] Liu M., Zhang Z., Liu H., Wu T., Han B. (2020). Chem. Commun..

[cit20] Rogers K. A., Zheng Y. (2016). ChemSusChem.

[cit21] Dong Z., Yuan J., Xiao Y., Mao P., Wang W. (2018). J. Org. Chem..

[cit22] Mei Y. Y., Zhang S. P., Wang H., Jing S. X., Hou T., Pang S. S. (2020). Energy Fuels.

[cit23] Duan H., Dong J., Gu X., Peng Y. K., Chen W., Issariyakul T., Myers W. K., Li M. J., Yi N., Kilpatrick A. F. R., Wang Y., Zheng X., Ji S., Wang Q., Feng J., Chen D., Li Y., Buffet J. C., Liu H., Tsang S. C. E., O'Hare D. (2017). Nat. Commun..

[cit24] Córdova A., Afewerki S., Palo-Nieto C. (2020). Synthesis.

[cit25] Ko C. H., Park S. H., Jeon J. K., Suh D. J., Jeong K. E., Park Y. K. (2012). Korean J. Chem. Eng..

[cit26] Huang J. L., Dai X. J., Li C. J. (2013). Eur. J. Org. Chem..

[cit27] Chen Q., Kang H. Z., Liu X., Jiang K., Bi Y. F., Zhou Y. M., Wang M. Y., Zhang M., Liu L., Xing E. H. (2020). ChemCatChem.

[cit28] Ibrahim J. J., Reddy C. B., Fang X. L., Yang Y. (2020). Eur. J. Org. Chem..

[cit29] Herrmann J. M., Konig B. (2013). Eur. J. Org. Chem..

[cit30] Sun Z., Fridrich B., de Santi A., Elangovan S., Barta K. (2018). Chem. Rev..

[cit31] Yang X. M., Liang Y., Cheng Y. Y., Song W., Wang X. F., Wang Z. C., Qiu J. S. (2014). Catal. Commun..

[cit32] Kalutharage N., Yi C. S. (2015). J. Am. Chem. Soc..

[cit33] Nishikawa H., Kawamoto D., Yamamoto Y., Ishida T., Ohashi H., Akita T., Honma T., Oji H., Kobayashi Y., Hamasaki A., Yokoyama T., Tokunaga M. (2013). J. Catal..

[cit34] Wang S. G., Zhou P., Jiang L., Zhang Z. H., Deng K. J., Zhang Y. H., Zhao Y. X., Li J. L., Bottle S., Zhu H. Y. (2018). J. Catal..

[cit35] Pei X., Deng Y., Duan B., Chan T.-S., Lee J.-F., Lei A., Zhang L. (2018). Nano Res..

[cit36] Besson M., Gallezot P., Pinel C. (2014). Chem. Rev..

[cit37] Antil N., Kumar A., Akhtar N., Newar R., Begum W., Manna K. (2021). Inorg. Chem..

[cit38] Zhang P., Chen N., Chen D., Yang S., Liu X., Wang L., Wu P., Phillip N., Yang G., Dai S. (2018). ChemCatChem.

[cit39] Sitthisa S., Pham T., Prasomsri T., Sooknoi T., Mallinson R. G., Resasco D. E. (2011). J. Catal..

[cit40] Wang H. F., Chen L., Pang H., Kaskel S., Xu Q. (2020). Chem. Soc. Rev..

[cit41] Gu Y., Wu Y. N., Li L., Chen W., Li F., Kitagawa S. (2017). Angew. Chem., Int. Ed..

[cit42] He H., Li R., Yang Z., Chai L., Jin L., Alhassan S. I., Ren L., Wang H., Huang L. (2021). Catal. Today.

[cit43] Lee J. G., Yoon S., Yang E., Lee J. H., Song K., Moon H. R., An K. (2020). J. Catal..

[cit44] Gong W., Lin Y., Chen C., Al-Mamun M., Lu H. S., Wang G., Zhang H., Zhao H. (2019). Adv. Mater..

[cit45] Zhong G. H., Liu D. X., Zhang J. Y. (2018). J. Mater. Chem. A.

[cit46] Kramm U. I., Herrmann-Geppert I., Behrends J., Lips K., Fiechter S., Bogdanoff P. (2016). J. Am. Chem. Soc..

[cit47] Jiang L., Zhou P., Liao C., Zhang Z., Jin S. (2018). ChemSusChem.

[cit48] Ranaware V., Verma D., Insyani R., Riaz A., Kim S. M., Kim J. (2019). Green Chem..

[cit49] Hou W. X., Huang Y., Liu X. (2020). Catal. Lett..

[cit50] Wei Z., Wang J., Mao S., Su D., Jin H., Wang Y., Xu F., Li H., Wang Y. (2015). ACS Catal..

