# Supplementary material for: Highly effective and chemoselective hydrodeoxygenation of aromatic alcohols
Source: Chem Sci. 2022 Jan 19;13(6):1629–35. doi: 10.1039/d1sc06430d (PMC8827088; doi:10.1039/d1sc06430d)
Supplement: SC-013-D1SC06430D-s001 [file SC-013-D1SC06430D-s001.pdf]

Electronic Supplementary Material (ESI) for Chemical Science.
This journal is © The Royal Society of Chemistry 2022

Electronic Supplementary Information for

Highly Effective and Chemoselective Hydrodeoxygenation of Aromatic Alcohols

Caiyun Xu,[a] Haihong Wu,*[a] Zhanrong Zhang,*[b] Bingxiao Zheng,[a] Jianxin Zhai,[a] , Kaili Zhang,[a] Wei Wu,[a] Xuelei Mei,[a] Mingyuan He,*[a] and Buxing Han*[a,b]

[a]*Shanghai Key Laboratory of Green Chemistry and Chemical Processes, School of Chemistry and Molecular Engineering, East China Normal University, Shanghai 200062, China. E-mails: hhwu@chem.ecnu.edu.cn; hemingyuan@126.com; hanbx@iccas.ac.cn*

[b]*Beijing National Laboratory for Molecular Sciences, CAS Key Laboratory of Colloid and Interface and Thermodynamics, CAS Research/Education Center for Excellence in Molecular Sciences, Institute of Chemistry, Chinese Academy of Sciences, Beijing 100190, China. E-mails: zhangzhanrong@iccas.ac.cn; hanbx@iccas.ac.cn*

Experimental

Materials. All the chemicals were provided by commercial suppliers and used as received without further purification, unless otherwise noted. Cobalt (II) nitrate hexa-hydrate ($Co(NO_3)_2·6H_2O$, ≥98%), 1-phenylethanol (98%), cobaltosic oxide (99.5%), 1-phenyl-1-propanol (98%), 4-methylbenzyl alcohol (99%), 2-methylbenzyl alcohol (98%), furfural, (99%), n-butanol (99.5%), n-pentanol (99%), n-hexanol (99%) and n-octanol (99%) were purchased from Innochem. Activated carbon was obtained from Ketjenblack (Japan). Zinc nitrate hexa-hydrate ($Zn(NO_3)_2·6H_2O$, ≥99%), ethyl alcohol (99.8%), methanol (99.9%), tetrahydrofuran (99%), cyclohexane (≥99.5%) were purchased from Sinopharm Chemical Reagent Co., Ltd. (Shanghai, China). 2-Methylimidazole (98%), n-dodecane (99%) were purchased from Acros. Ethyl benzene,1,10-Phenanthroline, (S)-(−)-α−methyl-2-naphthalenemethanol, 4-ethylbenzyl Alcohol (>98%), 4-methylbenzhydrol (>98%) were obtained from Aladdin. 1-(4-Methylphenyl)ethanol, (97%), 1-Phenyl-1-pentanol (95%), 1-(4-biphenylyl)ethanol (98%), 1-(4-ethoxyphenyl)ethanol (98%), 2-phenyl-2-pentanol (98%) were supplied by Alfa. 1-(3-Methoxyphenyl)ethanol, 1-(2-methylphenyl)ethanol were purchased from Accela. 1-(4-Aminophenyl)ethanol was obtained from Adamas. (S)-1-(Naphthalen-1-yl)ethanol, 1-(4-methoxyphenyl)ethanol, (2,4-dimethylphenyl)methanol (97%), 5-methyl-2-furaldehyde (98%), alcohol were purchased from Ark. 4-Isopropylbenzyl alcohol, vanillin(>98%) 4-hydroxy-3-methoxy-alpha-methylbenzyl alcohol were purchased from TCI.

Synthesis of catalyst. The synthesis of ZIF was conducted according to a previously reported method with some modifications.[1] Specifically, $Co(NO_3)_2·6H_2O$ (1.164 g, 4 mmol) was dissolved in 60 mL methanol with stirring to form a clear solution. The solution was then added to a 20 mL solution of 2-methylimidazole (1.968 g, 24 mmol) in methanol. After vigorous mixing, the solution was kept at room temperature for 24 h. The as-obtained precipitates were centrifuged and washed with ethanol several times, and then dried at 80 °C for 12 h. The produced Co-ZIF was designated as ZIF-67. Then, the ZIF-67 powder was calcinated at 500, 600 and 700 °C under a 10% H_2 /Ar atmosphere for 5 h. After reduction, Co@CN was produced from ZIF-67 and stored in a vacuum desiccator. The zinc-doped catalyst Zn-ZIF was prepared using the same method, except $Zn(NO_3)_2·6H_2O$ was used as Zn precursor.

The Co@CN-ph catalyst was also prepared by the conventional incipient wetness impregnation method. $Co(NO_3)_2·6H_2O$ (0.582 g, 2 mmol) was dissolved in 60 mL methanol while stirring to obtain a clear solution. Then 1,10-phenanthroline (2.162 g, 12 mmol) was dissolved in 20 mL of methanol, and the final mixture was stirred for 24 h, and then the solvent used for impregnation was evaporated. Finally, the dried powder was activated under

a 10% H_2 /Ar atmosphere for 5 h at 700°C, to afford the Co@CN-ph catalyst.

The Co@AC catalyst was prepared by a conventional incipient wetness impregnation method. Specifically, $Co(NO_3)_2{\cdot}6H_2O$ (0.36 g, 2 mmol) was dissolved in 20 mL ethanol under stirring to achieve a clear solution. Then 0.2 g of activated carbon (Ketjenblack, Japan) was transferred into the solution, and the final mixture was stirred at 80 °C for 24 h, and then the solvent used for the impregnation was evaporated. Finally, the Co@AC catalyst was synthesized by activating the dried powder at 700 °C under a 10% H_2/Ar flow for 5 h.

For the ZIF-67(AIR) catalyst, the ZIF-67 precursor was synthesized in the same way to that of Co@CN. Then the ZIF-67 was calcined in a muffle furnace under air atmosphere for 5 h. The temperature was ramped from room temperature to 700 °C at 5 °C min^{-1}. The synthesized catalyst was stored in a vacuum desiccator.

Catalyst characterization. The morphology and size of ZIF-67 and its reduced product (catalyst) were measured with Hitachi S-4800 scanning electron microscope (SEM) and JEOL JEM-2100 transmission electron microscope (TEM) at an accelerating voltage of 200 kV. The high angle annular dark- field scanning TEM (HAADF-STEM) were operated at 300 kV by a JEOL Grand ARM 300F. X-ray diffraction (XRD) patterns were collected on a Rigaku Ultima IV X-ray diffractometer using Cu Kα radiation at 35 kV and 25 mA (λ = 1.5405 Å) over a 2θ ranging from 5° to 90° at a scanning speed of 5°/min. X-ray photoelectron spectroscopy (XPS) was carried out using Al kα (hv = 1486.36 eV) radiation with a Thermo Scientific K-Alpha spectrometer. Nitrogen sorption isotherms were obtained at 77 K by using a BEL-SORP-MAX instrument after degassing the samples for 8 h under vacuum at 473 K. The specific surface area was measured using the Brunauer–Emmett–Teller (BET) analysis method.

Hydrodeoxygenation (HDO) reaction and product analysis. The HDO reaction of 1-phenylethanol was carried out in a Teflon-lined stainless steel reactor of 15 mL with mechanical stirring at approximately 800 rpm. In a typical experiment, 40 mg catalyst, 0.122g 1-phenylethanol (1 mmol), 2 mL ethanol and 1 mmol internal standard (n-dodecane) were added into the reactor. The autoclave was purged three times with H_2 (1 MPa), and sealed under a H_2 atmosphere (2 MPa). After 8 h reaction, the stirring was stopped, and the reactor was quenched in an ice water bath immediately. Then the reaction mixture was filtered through nylon membrane (Whatman, Φ13 mm×0.45 μm) to separate the liquid phase from the catalyst. Other HDO reactions were carried out in the same way.

Characterization methods: The qualitative analysis of products was conducted using GC-MS (Agilent 7890A, equipped with a mass detector) and by comparing with authentic samples. The conversion and yields of products were quantitatively analyzed using GC (Agilent 8890, equipped with a hydrogen flame-ionization detector, full electric pneumatic control, 280°C) based on internal standard curves and areas of integrated peak area.

In order to test the recyclability of Co@CN-700, the spent catalyst was separated from the reaction mixture using an external magnetic field. Then the recovered catalyst was washed with ethanol for 5 times, followed by drying in an oven at 60°C.

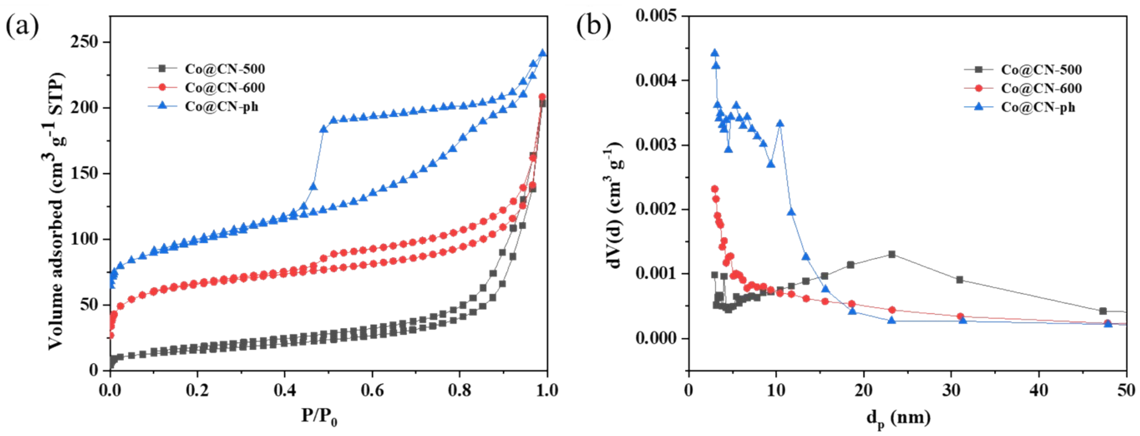

Fig. S1 (a) N_2-Adsorption-desorption isotherm of the Co@CN catalyst, (b) BJH pore size distribution of the Co@CN catalyst.

Table S1. Textural properties of the synthesized catalysts

Entry	Catalyst	Surface Area (m^2 g^{-1})	Pore Volume (cm^3 g^{-1})
1	Co@CN-ph	112.350	0.255
2	Co@CN-500	48.880	0.310
3	Co@CN-600	48.613	0.235
4	Co@CN-700	11.880	0.533

Table S2. ICP – OES analysis of the synthesized catalysts

Entry	Catalyst	Theoretical metal load of Co[a] (mmol)	Experimental metal load of Co[b] (wt. %)
1	Co@CN-ph	2	33
2	Co@CN-500	4	31.6
3	Co@CN-600	4	33.4
4	Co@CN-700	4	38.5

[a] Metal loading prior to calcination. The ratio of the amount of the metal to the ligand substance in the four materials is the same [b] Analyzed by ICP-OES.

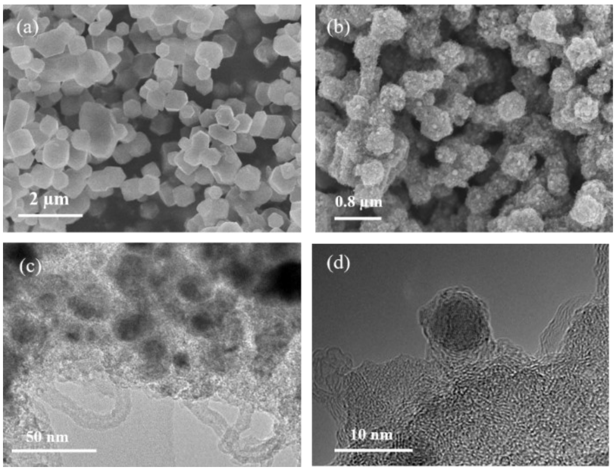

Figure. S2 (a) SEM image of the Co-ZIF, (b) SEM image of the Co@CN-700 catalyst, (c) and (d) HR-TEM images of Co@CN-700 catalyst.

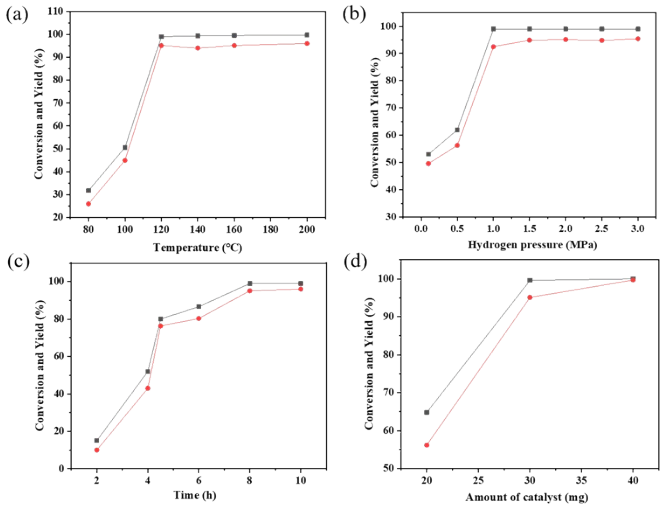

Figure. S3 (a) The effect of reaction temperature, (b) hydrogen pressure, (c) reaction time and (d) catalyst loading on the HDO reaction of 1-phenylethanol. Other reaction conditions are consistent with the optimal conditions: 1-phenylethanol (1 mmol), ethanol (2 mL), catalyst: Co@CN-700(40 mg), H_2: 2 MPa, temperature: 120 °C, time: 8 h, internal standard: n-dodecane (1 mmol).

Stability test of ethanol and HDO reaction of aliphatic alcohols

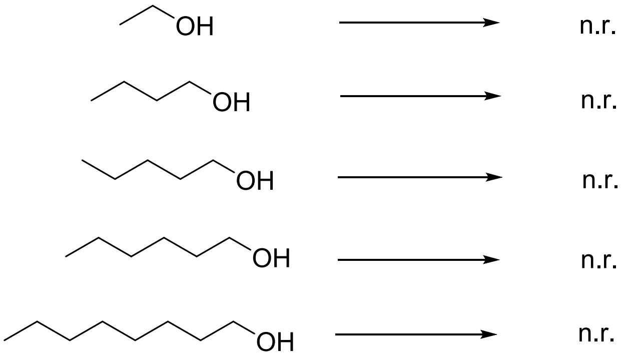

Figure. S4 Reaction conditions were the same to those in Table 1, entry 1: substrate (1 mmol), ethanol (2 mL), catalyst: Co@CN-700 (40 mg), H_2: 2 MPa, temperature: 120 ℃, time: 8 h, internal standard: n-dodecane (1 mmol), n.r. means no reaction.

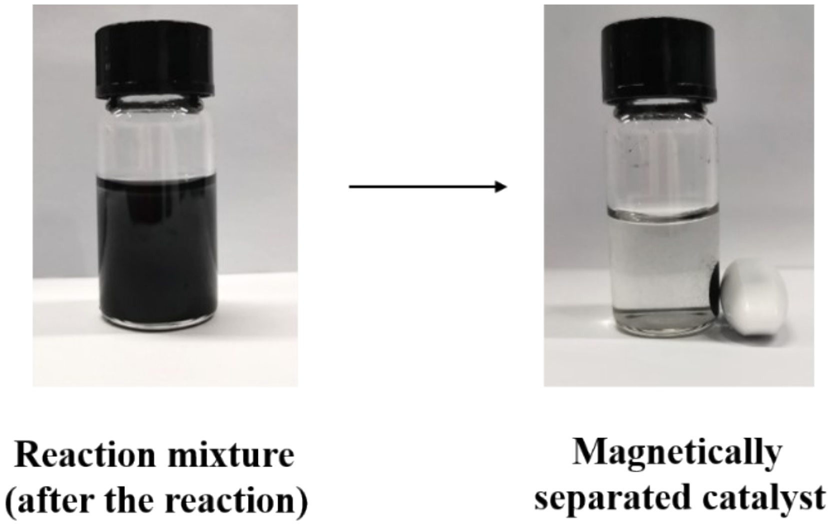

Figure. S5 Recovery of catalyst.

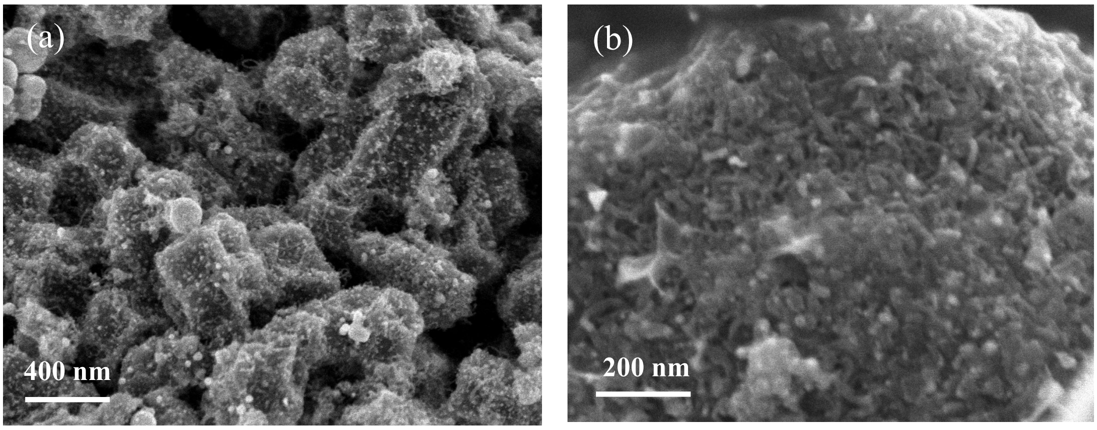

Figure. S6 SEM images of the spent Co@CN-700 catalyst.

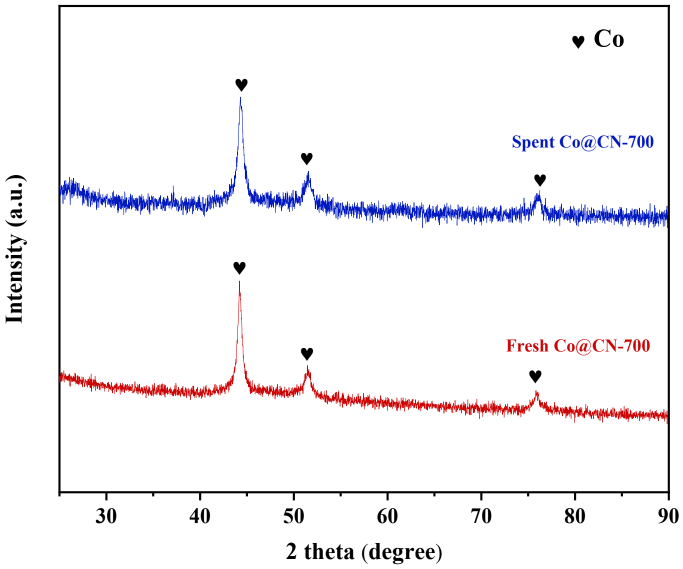

Fig. S7 XRD patterns of the used Co@CN-700 catalyst after 5 consecutive runs (upper image, blue) and before reaction (lower image, red).

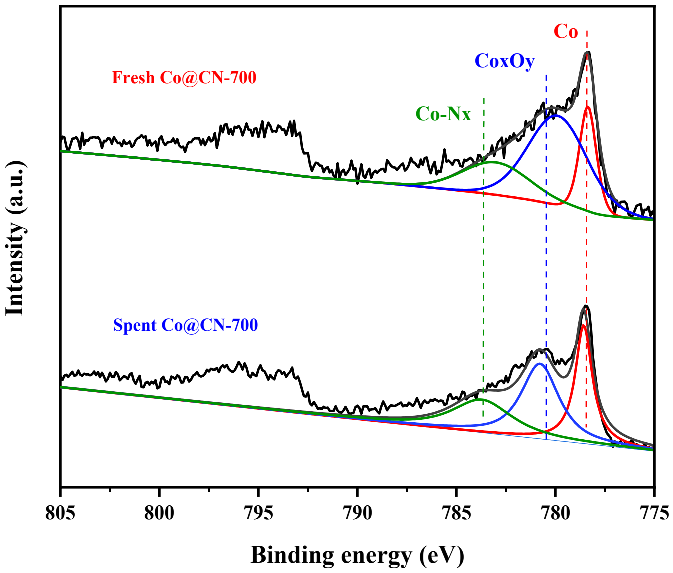

Fig. S8 XPS spectra of Co@CN-700 catalyst before (above) and after (below) reaction.

Reference

1. V. Ranaware, D. Verma, R. Insyani, A. Riaz, S. M. Kim and J. Kim, *Green Chemistry*, 2019, **21**, 1021-1042.
